# NMR Metabonomic Profile of Preterm Human Milk in the First Month of Lactation: From Extreme to Moderate Prematurity

**DOI:** 10.3390/foods11030345

**Published:** 2022-01-26

**Authors:** Chiara Peila, Stefano Sottemano, Flaminia Cesare Marincola, Matteo Stocchero, Nicoletta Grazia Pusceddu, Angelica Dessì, Eugenio Baraldi, Vassilios Fanos, Enrico Bertino

**Affiliations:** 1 Neonatal Unit, University of Turin, City of Health and Science of Turin, 10126 Turin, Italy; chiara.peila@unito.it (C.P.); stefano.sottemano@unito.it (S.S.); enrico.bertino@unito.it (E.B.); 2 Department of Chemical and Geological Sciences, Cittadella Universitaria di Monserrato, University of Cagliari, Monserrato, 09042 Cagliari, Italy; nicoletta.labor@gmail.com; 3 Department of Women’s and Children’s Health, University of Padova, 35128 Padova, Italy; eugenio.baraldi@unipd.it; 4 Institute of Pediatric Research (IRP), Fondazione Città della Speranza, 35128 Padova, Italy; 5 Neonatal Intensive Care Unit, Neonatal Pathology and Neonatal Section, Azienda University Polyclinic, University of Cagliari, 09042 Cagliari, Italy; angelicadessi@unica.it (A.D.); vafanos@tin.it (V.F.)

**Keywords:** metabolomics, NMR, human milk, preterm infant, gestational age

## Abstract

Understanding the composition of human milk (HM) can provide important insights into the links between infant nutrition, health, and development. In the present work, we have longitudinally investigated the metabolome of milk from 36 women delivering preterm at different gestational ages (GA): extremely (<28 weeks GA), very (29–31 weeks GA) or moderate (32–34 weeks GA) premature. Milk samples were collected at three lactation stages: colostrum (3–6 days post-partum), transitional milk (7–15 days post-partum) and mature milk (16–26 days post-partum). Multivariate and univariate statistical data analyses were performed on the ^1^H NMR metabolic profiles of specimens in relation to the degree of prematurity and lactation stage. We observed a high impact of both the mother’s phenotype and lactation time on HM metabolome composition. Furthermore, statistically significant differences, although weak, were observed in terms of GA when comparing extremely and moderately preterm milk. Overall, our study provides new insights into preterm HM metabolome composition that may help to optimize feeding of preterm newborns, and thus improve the postnatal growth and later health outcomes of these fragile patients.

## 1. Introduction

Human milk (HM) is the best way to feed a newborn in the first months of life as it contains a balanced amount of nutrients and bioactive components which guarantee optimal growth and development of the organs, immune system, and intestinal microbiota. Furthermore, breastfeeding is associated with several short- and long-term positive health effects for both infant and mother. Short-term benefits for the infant concern defense against gastrointestinal and respiratory infections and atopic diseases [1]. Long-term benefits include the reduction of the prevalence of overweight/obesity and type 2 diabetes [1]. Furthermore, the health effects experienced by the mother on a short-term scale include a decreased risk of iron-deficiency anemia, improvements in mother-baby bonding, and prevention of postpartum hemorrhage. The long-term benefits include decreased risk of breast cancer, ovarian cancer, type 2 diabetes, hypertension, hyperlipidemia, and cardiovascular diseases [2].

The composition of HM is influenced by many factors including gestational age (GA), lactation stage, the mother’s body mass index (BMI), parity number, diurnal variations, and maternal diet [3,4]. Despite the extended evidence of the inter- and intra-variability of HM composition, its dynamic nature, and complexity as well as the potential interactions among milk components continue to limit our understanding of the functional power of HM. This knowledge is of relevance in the management of infant feeding and extremely important in case of fragile subjects such as preterm newborns which, due to their condition of prematurity, are at high-risk for short and long-term complications [5,6].

In addition to the variability of HM composition in terms of macronutrients, during the past decade, attention has been turned toward the HM metabolome, that is the pool of low molecular weight (<1.5 kDa) metabolites associated with gene expression and protein activity. This class of compounds includes mainly free amino acids, organic acids, carbohydrates, HM oligosaccharides (HMOs), and other metabolites important for infant development, such as choline and creatine. Metabolomics studies on HM have pointed out compositional variations in the metabolome related to such maternal characteristics as genetics, diet, and lifestyle, as well as gestation age (terms vs. preterm delivery), type of delivery, lactation time, and geographical location [7,8,9,10,11,12,13,14,15,16,17,18,19,20,21]. For more detailed information on this topic, the reader is directed to two recent reviews [22,23]. Nevertheless, despite the rapid progress in knowledge of HM metabolome composition, its characterization remains incomplete, and its role in the infant nutrition is not totally understood.

The aim of the present study was to contribute to the advance of biological knowledge of the composition of the preterm HM metabolome. In particular, by using a metabolomics approach based on Nuclear Magnetic Resonance (NMR) spectroscopy, we analyzed and compared the metabolic profiles of HM samples collected in the first month post-partum from three groups of mothers (*n* = 36) delivering extremely (*n* = 14), very (*n* = 11) and moderately (*n* = 11) premature infants. Compositional variability was analyzed in terms of the degree of prematurity and the lactation stage.

## 2. Materials and Methods

### 2.1. Study Population

Thirty-six women delivering preterm infants were recruited at the University, City of Health and Science of Turin. Informed consent was obtained from all subjects at enrolment. Mothers with major morbidities (diabetes mellitus, preclampsia or eclampsia) or with absence or interruption of breastfeeding during newborn admission to the Neonatal Unit were excluded. This study was conducted in accordance with the declaration of Helsinki and approved by the local ethics committee (n.0039644).

### 2.2. Sample Collection

Human milk samples were collected from each mother within the first month of exclusive breast-feeding at three lactation times. The first sample was colostrum (from 0 to 6 days post-partum), the second was considered transitional (from 7 to 15 days post-partum) and the third was mature milk (from 16 to 26 days post-partum). Milk was collected with standard extraction methods before baby feeding by means of an electric breast pump (Medela Symphony, Baar, Switzerland) at hospital in the morning from one breast into a sterile polypropylene tube. According to current guidelines and in order to collect full pumping samples, the extraction session was stopped 2 min after the outflow of the last drops of milk [24,25]. Samples were stored at −80 °C and shipped on dry ice to the University of Cagliari for NMR analysis.

### 2.3. Sample Preparation

Before ^1^H NMR analysis, milk samples were thawed in ice. An aliquot of 500 μL was centrifuged at 10,000× *g* for 30 min at 4 °C using Amicon Ultra 0.5 mL 10 kDa spin filters (Millipore, Billerica, MA, USA) in order to remove residual lipids and proteins. All filters were extensively washed with distilled water to deprive the membrane of the embedded glycerol. The procedure was carried out iteratively until control by NMR spectroscopy of the wash water showed no residual presence of glycerol. Each filtered sample (350 μL) was mixed with 250 μL of phosphate buffer (0.1 M, pH 7.4) in D_2_O and 50 μL of 30 mM sodium 3-trimethylsilyl-(2,2,3,3-^2^H_4_)-1-propionate (TSP) internal standard solution (in D_2_O) and then transferred into a 5 mm wide NMR tube.

### 2.4. ^1^H NMR Spectroscopy and Spectral Processing

^1^H NMR experiments were performed at 300 K on a Varian UNITY INOVA 500 spectrometer (Agilent Technologies, Inc., Santa Clara, CA, USA), operating at a frequency of 499.83 MHz. One-dimensional (1D) ^1^H NMR spectra were obtained using a standard pulse sequence (1D NOESY) with presaturation during relaxation and mixing time for water suppression. For each milk spectrum, a total of 128 scans were collected in 64 k data points over a spectral width of 6000 Hz using a recycle time of 3.5 s and a mixing time of 1 ms. After Fourier transformation with 0.3 Hz line broadening, the spectra were phased and baseline corrected and the chemical shift scale was set by assigning a value to the signal of TSP of δ = 0.00 ppm.

NMR spectra were processed using MestReNova, version 14.0 (Mestrelab Research SL, Santiago de Compostela, Spain) and corrected for misalignments in chemical shift primarily due to pH-dependent signals. Each spectrum was binned using a constant bin of 0.001 ppm in the region between 9.00 and 0.75 ppm, excluding peaks from water (4.6−5.2 ppm). The final data set was composed of 7650 features.

The annotation of the ^1^H NMR spectra was based on literature data [18,20,26] and the Human Metabolome Database (https://hmdb.ca, accessed on 7 September 2021). If available, the spectra of standard compounds recorded using the same experimental conditions were compared with the experimental data for identification. As a result, 34 metabolites were assigned to the spectra. Their signals were annotated with MSI Level 1 or Level 2 [27] and manually integrated obtaining a data set composed of 44 features. Probabilistic Quotient Normalization was applied to compensate for dilution effect.

### 2.5. Statistical Data Analysis

Data of the recruited mothers and neonates were investigated applying one-way ANOVA for normally distributed data and Fisher’s exact test or the Chi-squared test for categorical data with two or more than two levels, respectively; tests with *p* less than 0.05 were considered statistically significant. Normality was assessed using the Shapiro-Wilk test assuming normally distributed data for *p* > 0.10.

Exploratory analysis of the metabolomics data was performed by Principal Component Analysis (PCA). PCA is able to summarize the structured data variation of correlated, redundant and noisy data using a small number of score vectors, called principal components (PCs), obtained by linear combination of the measured features. As a result, model interpretation based on suitable plots allows the investigation of complex data structures of the discovery of patterns in the observations and the relationships between observations and measured features.

To evaluate the effects of lactation stage and degree of prematurity on the metabolic content of the milk, we applied both univariate and multivariate data analyses. Specifically, the lactation stage represented using the post-partum day of milk sample collection (factor time) and the degree of prematurity (factor prematurity with three levels: extremely, very and moderately preterm delivery), were codified as quantitative multilevel factors and by sum coding [28], respectively, and were included in the design matrix of the fixed effect X_fixed_ for both univariate and multivariate modelling. In the case of univariate data analysis, linear mixed effects (LME) modelling for longitudinal data [29] was applied while controlling for the false discovery rate using the Benjamini-Hochberg procedure [30]. The following model was considered:

y_ij_ = (b_0_ + u_0i_) + (b_1_ + u_1i_) time_ij_ + b_2_ prematurity_i_ + ε_ij_

where y_ij_ is the value of the feature y measured in the milk sample of the mother i collected at time j, time_ij_ is the post-partum day of sample collection, prematurity_i_ is the degree of prematurity of the newborn of mother i, b_k_ with k = 0, 1, 2 are the coefficients of the fixed effects, u_0i_ and u_1i_ are the random effects describing the specific-mother effects and ε_ij_ is the random error. The model assumes that the growth curves show a similar pattern across mothers (a linear trend is assumed); however, important individual differences may be exhibited in both the intercept and the slope, as typically occurs with longitudinal data.

Multivariate data analysis was performed using a new model developed combining LME and Partial Least Squares regression (PLS2) [31]. This approach is inspired by APCA+ [32], a multivariate technique useful for investigating data generated considering independent observations and a well-defined experimental design; unfortunately, this method is not suitable for longitudinal data as the observations are closely related. Given the matrix of the measured features, Y, and the design matrix of the fixed effects X_fixed_, the objective is to decompose Y as

Y = Y_fixed_ + Y_random_ + F

where the data variation in Y_fixed_ is explained by X_fixed_, the data variation in Y_random_ is associated with the random effects described by a suitable design matrix X_random_, and the matrix F is the part of Y that is not explained by the design matrices. The idea is to use PLS2 and LME to model Y_fixed_ and Y_random_, respectively. The two-step procedure described in the Supplementary Materials is used to solve the problem. As a result, the data variation associated with the fixed effects is modeled by the bilinear form

Y_fixed_ = TP^t^

where T is the score matrix and P the loading matrix, similarly to PCA. Thus, procrustes analysis was applied to transform the scores into latent factors. The investigation of the latent factor space allows the discovery of specific trends and cluster structures between observations, whereas stability selection based on Variable Influence on Projection (VIP) [33] permits the identification of the most relevant features. Repeated five-fold cross-validation and permutation testing was applied in order to highlight over-fitting and assess model reliability.

Data analysis was performed using in-house R-functions implemented by R 4.0.4 platform (R Foundation for Statistical Computing). The R-functions used in this study are available on request.

## 3. Results

### 3.1. Study Population

The characteristics of the mother and infant population are summarized in Table 1. Overall, 36 healthy lactating mothers giving birth prematurely between 23 and 33 weeks of gestation were recruited. According to the degree of prematurity, subjects were grouped into three groups: extremely, very, and moderately preterm delivery. No significant differences among the three groups were observed in the mother’s age and body mass index (BMI). The only relevant difference was in the delivery mode, which was mainly vaginal (71%) in the extremely preterm group and cesarian section (82%) in the other two groups. Regarding the newborns gender, there were 26 female and 18 male newborns with a birth weight ranging from 500 to 2250 g.

Of the 36 enrolled women, 25 provided three milk samples (colostrum, transitional milk and mature milk), while seven provided only colostrum and transitional milk, and four provided transitional and mature milk; 97 human milk samples (32 colostrum, 36 transitional milk and 29 mature milk) were ultimately analysed by ^1^H NMR spectroscopy.

### 3.2. Mother Phenotype

**Figure 1:**
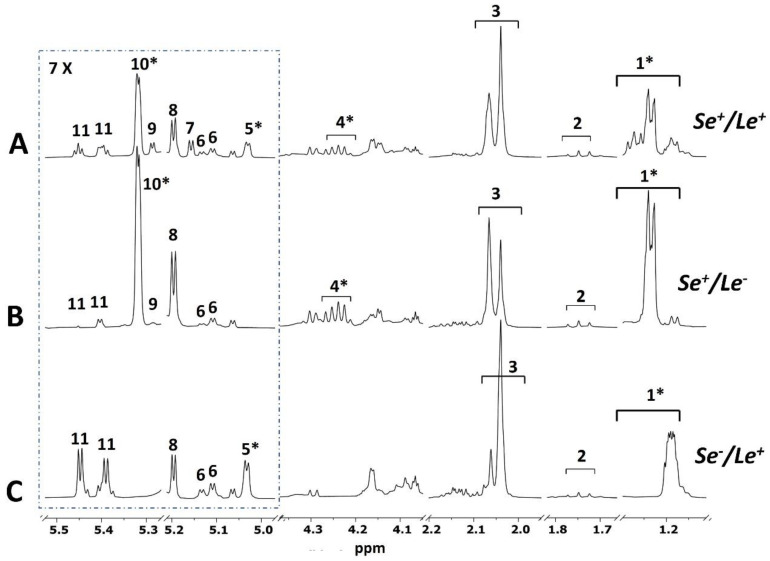
Expanses of representative 1D NOESY-presat ^1^H NMR spectra (500 MHz, 300 K) of preterm human milk (350 μL of filtered milk, 250 μL of 0.1 M PBS in D_2_O, pH 7.4 and 50 μL of 30 mM TSP solution in D_2_O) of three mother phenotypes: (**A**) Se^+^/Le^+^, (**B**) Se^+^/Le^−^, (**C**) Se^−^/Le^+^. Symbol * denotes HMOs signals taken into consideration for the identification of phenotype. Key: 1. Fuc CH_3_ (δ 1.14–1.30) in: α1,3- and α1,4-linked Fuc residues (δ 1.14–1.19), α1,2-linked Fuc residues (δ 1.19–1.24) and α1,2-Fuc with α1,4-Fuc and/or α1,2-Fuc with α1,3-Fuc (δ 1.24–1.30); 2. 3′ sialyllactose, 6′ sialyllactose; 3. *N*-Acetylglucosamine containing oligosaccharides; 4. Fuc H-5 in α1,2-linked Fuc residues; 5. Fuc H-1 in α1,4-linked Fuc residues; 6. Fuc H-1 in α1,3-linked Fuc residues; 7. Fuc H-1 in α1,2-linked Fuc residues (LNDFH I, lacto-*N*-difucohexaoses I); 8. Reducing α-Glc units; 9. Fuc H-1 in α1,2-linked Fuc residues (LDFT, lactodifucotetraose); 10. Fuc H-1 in α1,2-linked Fuc residues (2′FL, 2′fucosyllactose; LNFP I, lacto-*N*-fucopentaoses I); 11. Fuc H-1 in α1,3-linked Fuc residues.

Since the fucosylation patterns of HM oligosaccharides (HMOs) are influenced by the mother’s genotype, visual inspection of the HMOs’ NMR spectral regions allowed the identification of the phenotypic status of the mother [11,18,34]. In particular, based on the activity of specific enzymes called fucosyltransferase, involved in HMO synthesis, mothers can be classified for two types of genes: the secretory gene (Se), which encodes α1,2-fucosyltransferase (FucT2), and the Lewis gene of blood groups (Le), which encodes α1,3 (FucT3) and α1,4-fucosyltransferase (FucT4) [35]. The milk from secretor women (Se^+^) contains α1-2 fucosylated HMOs; conversely that from non-secretor women (Se^−^) contains no or only trace α1-2 fucosylated HMOs. The milk from Lewis-positive women (Le^+^) contains α1-4 fucosylated HMOs, while that from Lewis-negative (Le^−^) women contains no or minimal amounts of these HMOs. Based on the data in the literature [11,18,34], monitoring the presence or the absence of specific HMOs NMR peaks allowed us to assign milk samples to three phenotypes (Figure 1). Twenty-six women (72%) were classified as Se^+^/Le^+^ as their corresponding milk spectra displayed the CH_3_ signals from α1,2-, α1,3- and α1,4-linked fucosyl residues (Figure 1A). Four women (11%) were assigned to the Se^+^/Le^−^ phenotype, because their milk ^1^H NMR spectra exhibited the peaks from α1,2- and α1,3-linked fucosyl residues and lacked the characteristic doublet of α-1,4 residues in the range *δ* 5.00–5.05 (Figure 1B). Six women (17%) were classified as Se^−^/Le^+^, defined by lack of NMR signals from α1,2-fucosylated HMOs and the presence of signals from α1,3- and α1,4-fucosylated HMOs (Figure 1C). The number of phenotypes identified in each premature delivery group is reported in Table 1.

### 3.3. Exploratory Data Analysis

Exploratory data analysis was performed considering the binned spectra. Prior to performing data analysis, mean centering and Pareto scaling were applied. Outlier detection was based on principal component analysis (PCA) considering the T2 test and the Q test. Specifically, each subgroup of samples with the same degree of prematurity and lactation stage (colostrum, transitional milk, and mature milk) was submitted to PCA and the statistics T2 and Q were calculated for each sample. No outliers were detected when assuming a significance level of 0.05.

**Figure 2:**
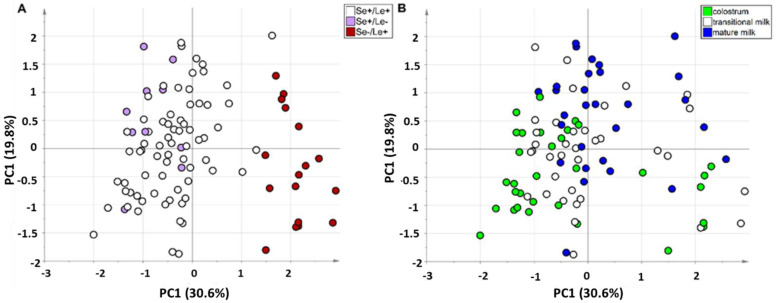
PC1 vs. PC2 score scatter plot of the PCA model built with the binned data, colored according to maternal HMO phenotype (panel (**A**): Se^+^/Le^+^ in white; Se^+^/Le^−^ in violet; Se^−^/Le^+^ in red) and lactation stage (panel (**B**): colostrum in green; transitional milk in white; mature milk in blue).

Due to the presence of multiple intense NMR signals from lactose dominating the spectrum, the peaks of this carbohydrate resonating between 3.50 and 4.05 ppm and between 3.26 and 3.34 ppm were excluded. The PCA model with two principal components showed R^2^ = 0.50 and Q^2^ = 0.47 (calculated by five-fold cross-validation based on the Krzanowski method). Projecting the observations on the plane spanned by the first two PCs provided the score scatter plot reported in Figure 2. In Figure 2A the observations are colored according to phenotype, while in Figure 2B they are colored according to lactation stage. A clear separation among samples of different phenotypes can be observed in Figure 2A. Specifically, samples from women phenotyped as secretors (Se^+^) are mainly distributed on the left part of the plot, while those from non-secretors (Se^−^) are located on the right one. Additionally, within the Se^+^ group a weak separation of samples is visible based on the Lewis phenotype, with the Se^+^/Le^−^ subgroup being slightly shifted toward the upper left quadrant with respect to the Se^+^/Le^+^ subgroup. Accordingly, the analysis of the PCA biplot indicated that such a score distribution is mainly driven by differences in the chemical components of the HMOs (Figure S1 in Supplementary Materials). A further contribution to the score distribution was explained in terms of the lactation stage. Indeed, a shift of scores from the left bottom side of the plot to the right upper one is visible based on increasing lactation stage (i.e., from colostrum to mature milk) (Figure 2B). No effect of the degree of prematurity was revealed.

### 3.4. Studying the Changes in HM Metabolome Due to Degree of Prematurity and Lactation Stage

The exploratory data analysis based on PCA highlighted an important impact of both lactation stage and the mother’s genetics on the metabolic content of HM. To assess whether the degree of prematurity influences the variation of the collected data, the experimental design was explicitly taken into account in data modelling. In the following, 44 features arising from peak annotation were considered. Data were autoscaled prior to performing data analysis.

As a first step in data analysis, all the three degrees of prematurity were considered. Neither univariate nor multivariate data analysis highlighted the prematurity effect as significant and only the influence of lactation stage on the data was discovered. Specifically, no features associated with prematurity were selected when controlling the false discovery rate at level δ = 0.15 by applying linear mixed effects (LME) modelling for longitudinal data. The multivariate model showed that the prematurity factor was not significant, being R^2^_prematurity_ = 0.41 (*p* = 0.80) and Q^2^_prematurity_ = 0.06 (*p* = 0.35). On the other hand, 34 features were associated with the time factor on the basis of LME analysis controlling the false discovery rate at level δ = 0.05 (Table 2). In addition, the multivariate model proved that lactation stage significantly affected data variation, showing R^2^_time_ = 0.74 (*p* = 0.01) and Q^2^_time_ = 0.46 (*p* = 0.02). These results agree with those of the above-mentioned exploratory data analysis based on PCA.

As a second step in the data analysis, only the two groups with the greatest differences in the degree of prematurity (i.e., extremely and moderately preterm) were considered. In that case, new and interesting results were obtained. No statistically significant differences were observed for mother’s age, BMI, type of pregnancy, or infant gender, while a relevant difference was observed for both delivery mode and infant birth weight (Table S1 in Supplementary Materials).

**Figure 3:**
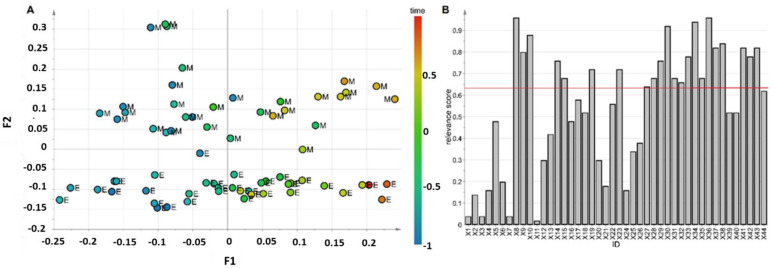
Multivariate data model of the data set arising from annotated spectra. Panel (**A**): Latent factor scatter plot colored according to time; M and E labels indicate milk samples from mothers delivering moderately and extremely preterm, respectively. Panel (**B**): Relevance score plot; the red line indicates the threshold at the level of α = 0.05 used for discovering relevant features. The meaning of the feature codes is reported in Table S2 in the Supporting Information.

**Figure 4:**
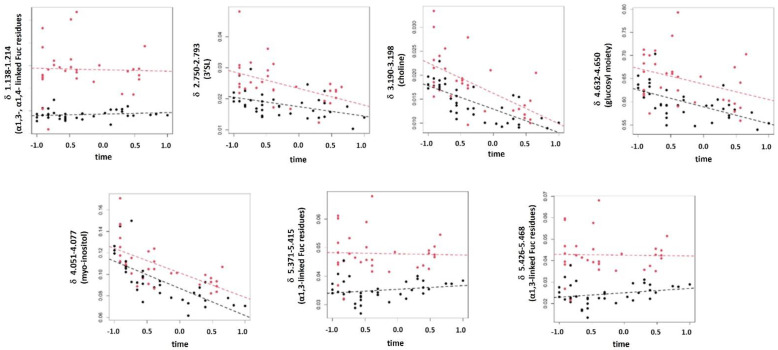
Scatter plots showing the trends of the sum of the fixed effect part and the random error of the LME models considering the seven relevant ^1^H NMR annotated signals influenced by the degree of prematurity. Red circles represent samples from mothers delivering moderately preterm, black circles represent samples from mothers delivering extremely preterm, and dashed lines represent the average behavior over time of the two groups.

The results of the LME analysis are reported in Table S2 and Figure S2 in the Supplementary Materials. Despite three features showing *p* < 0.05 for the prematurity factor, no signals could be considered significantly affected by the degree of prematurity when controlling the false discovery rate level at δ = 0.15. Moreover, 33 signals were significantly affected by time when controlling the false discovery rate level at δ = 0.05. On the other hand, the multivariate model showed three score components, R^2^_time_ = 0.76 (*p* = 0.01), Q^2^_time_ = 0.40 (*p* = 0.01), R^2^_prematurity_ = 0.80 (*p* = 0.01), Q^2^_prematurity_ = 0.43 (*p* = 0.06). After post-transformation, two latent factors (F1 and F2) were generated. The related scatter plot is reported in Figure 3A. The first latent factor, F1, was mainly associated with time, whereas the second latent factor, F2, was explained by prematurity. In particular, the group of samples from mothers delivering extremely preterm belonged to the region with negative values of F2, while the samples of the other group belonged to the region with positive values, independent of the time. Stability selection based on VIP highlighted 22 features as relevant (Figure 3B) among which seven were influenced by the degree of prematurity. In particular, as shown in Figure 4, for both groups, the contents of α1,3-linked Fuc residues were almost independent of lactation stage, whereas the levels of the 3′SL, choline, myo-inositol, and glucosyl moieties decreased with time. Furthermore, the concentration of these metabolites was higher in the samples from mothers delivering moderately preterm than in those from women delivering extremely preterm at the same lactation stage.

## 4. Discussion

For a preterm (PT) infant, optimal nutrition is a determining condition of a general and neurological development. Indeed, by losing the terminal part of pregnancy in the intrauterine environment, PT newborns miss the most critical period of development and brain growth, and are, thus, at high risk of short- and long-term morbidities as immature physiology, hypothermia, respiratory distress, apnea, hypoglycemia, and developmental delays [5]. According to the recommendation of the American Academy of Pediatrics [6], the postnatal growth of PT infants, referred to anthropometric indices and body composition, should be the same as a normal fetus of the same gestational age growing in its mother’s uterus. Currently, the mother’s own milk is considered the “gold standard” nutrition for feeding pre-term newborns, as fresh human milk (HM) or banked HM cannot meet nutritional recommendations for these fragile subjects (especially for extremely low birth weight newborns) [36,37]. Thus, the addition of fortifiers to expressed HM is commonplace to meet recommended intakes, mainly in the form of protein, calcium, phosphorus, carbohydrate, vitamins, and minerals [38]. The widely used standard fortification method assumes a uniform composition of HM irrespective of maternal characteristics, gestation, and day of lactation, and applies a fixed dose of nutrients to milk. This method rarely meets the recommended intake of nutrients for preterm infants, which can create the conditions for risk of under- or over-nutrition. Recently, favorable results have been obtained with individual fortification of HM in an attempt to compensate for the high variability of expressed breast milk composition [39]. Nevertheless, despite the international nutritional management recommendations in hospital and after discharge from hospital [40,41], PT newborn nutrition remains one of the most complex tasks for neonatologists. In this respect, an important support in decision making on optimal infant nutrition arises from the characterization of HM composition, a fundamental prerequisite for understanding its biological functions.

In addition to milk macronutrients, in recent years special attention has been paid to another component of HM, called the metabolome, which includes a wide class of low molecular weight (<1.5 kDa) molecules such as carbohydrates, nonprotein nitrogen molecules, polyamines, HMOs, choline derivatives, organic acids, and some vitamins [22,23]. In particular, the evidence for inter- and intra-variability of the HM metabolome composition associated with both genetic and nongenetic-factors has suggested an important role of this molecular component in the programming of neonates, with potentially relevant contributions to the growth and neurodevelopmental of infants. Most of the metabolomics studies on HM so far have been performed on term milk, while a lower number have been conducted on preterm milk.

The present study aimed to deepen knowledge of the metabolome of milk from mothers with preterm delivery by investigating compositional variability in terms of both lactation stage over the first month after birth and prematurity degree. It is worth restating that the impact of lactation time on HM has been already investigated in other metabolomics studies which concluded that significant variations exist in the metabolome composition over time [15,16,21,26,42]. The influence of prematurity has been previously pointed out as well, although this was confined to a comparison between full term and preterm birth [14,15]. Nevertheless, because of different experimental designs, the findings in the literature remain in some respects inconsistent, thus making direct comparisons complicated. Compared to the metabolomics literature on preterm HM [15,16,26], our experimental design is characterized by a higher number of enrolled mothers, a more complete longitudinal collection of samples (80% of women provided specimens at the three lactation stages under investigation) and a division of samples into three subcategories according to gestational age (extremely, very and moderately preterm delivery). The unsupervised analysis of our whole NMR data set captured a principal and remarkable inter-variability of preterm HM metabolome driven by differences in HMO components and a secondary significant intra-variability over lactation in the levels of various metabolites. These characteristics are broadly in agreement with ththose reported in the literature. Indeed, a variability of the NMR spectral profile of metabolome in the first month of lactation was observed by Spevacek et al. [16] primarily in terms of lactation stage and secondarily in terms of genetic factors, while no significant difference in clustering between milk from mothers delivering term and preterm infants was noted. Sundekilde et al. [15] pointed out a great contribution to HM metabolome variation by both gestational age (term vs. preterm birth) and lactation stage. Furthermore, over a temporal window of three months after birth, Andreas et al. [21] evidenced dynamic changes in the levels of various metabolites in the HM metabolome by using a multiplatform approach.

In this study, we observed a significant temporal change in the levels of certain free amino acids (FAAs) as the lactation stage progressed from colostrum to mature human milk. In particular, alanine, glutamate and glutamine significantly increased, while leucine and aspartate significantly decreased. These findings closely mirror previously published data [43]. FAAs of HM are more readily absorbed than protein-derived amino acids. Although they comprise approximately 5% of the total AA content in human milk, FAAs participate in a number of metabolic processes as precursors of the biosynthesis of numerous important biological and physiological compounds [43]. Glutamine and glutamate, together with taurine, make up 50% of the total FAAs in HM. They are proposed to play an important role in enhancing immune function. Glutamate is a signaling molecule involved in sustaining gut barrier function and neuroendocrine reflexes. Furthermore, it is known as an appetite regulator and may act as a neurotransmitter in the brain. Glutamine is a nonessential amino acid that supplies ketoglutaric acid for the citric acid cycle and serves as a brain neurotransmitter. Leucine is an amino acid extensively evaluated for its ability to enhance muscle protein synthesis in low protein diets, making it an ideal candidate for stimulating growth of low birth weight infants [44]. A previous characterization of the PT HM metabolome has indicated no significant changes in most FAAs during the first month of lactation, while the exception of an increase in alanine [16]. It is likely that the different result here reported is due to the greater number of HM specimens analyzed in the present report, allowing for a higher power of statistical analysis.

Furthermore, earlier metabolomics findings have indicated that the levels of most sugars in the PT milk metabolome vary over lactation [15,16], although no significant change in the first month postpartum was observed except for the increase of lactose [16]. In our study, however, in addition to the significant increase in lactose level, we observed a significant decrease in the content of α1,2-linked fucosyl HMOs and sialyllactoses, while no significant variations of α1,3- and α1,4-linked fucosyl residues were found. The behavior of α1,2-fucosylated HMOs suggests a reduced activity of the secretor FucT2 enzyme during the course of lactation, which is in good agreements with the literature [45,46]. Similarly, the decreasing trend of sialyloligosaccharide levels during the first month of lactation parallels that already reported for both preterm [45,46] and term [45,46,47,48] milk. In addition, the absence of a significant variation in α1,4-linked fucosyl HMOs mirrors the data in the literature [45]. Unlike our findings, in several previous studies [45,46,47,48,49,50], the level of α1,3-fucosylated oligosaccharides increased during lactation. Although this contrasts with our results, it is worth noting that the lack of standardized methods of analysis, milk collection timing, and sample preparation make it difficult to perform a direct inter-lab comparison. Therefore, at present, we can only speculate about the cause of the latter observation.

This study showed a statistically significant time-dependent decrease in choline, myo-inositol, and pantothenic acid in PT human milk. These metabolites are all important nutrients and crucial for infant growth and development. Choline plays a significant role in the continued growth of the brain and in cognitive measures [51]. It is a precursor of phosphocholine and sphingomyelin, two constitutive membrane phospholipids and water-soluble metabolites serving as osmoregulation and important methylation processes. In breast milk, phosphocholine and glycerophosphocholine are the most abundant choline carriers. The contents of choline compounds in mature breast milk vary considerably among breastfeeding women independent of the lactating period [52], probably representing one of the causes of conflicting evidence of the changes over lactation. Myo-inositol is the major stereoisomer of inositol in the body, with several important roles in the central nervous system [53]. Furthermore, it is a constituent of a number of inositol-phosphates, glycolipids, and glycoproteins, components of various membranous structures. Our data agree with previous publications, which showed that preterm colostrum has higher myo-inositol concentrations than mature preterm [54,55]. Pantothenic acid (vitamin B5) belongs to the B vitamin class of organic compounds provided mainly by dietary intake. B vitamins act as cofactors and coenzymes in several metabolic reactions, such as the citric acid cycle and one-carbon metabolism and are crucial for a developing infant. Preterm infants, in particular, are at high risk of vitamin deficiencies due to limited stores at birth, as well as to increased needs related to their rapid growth and development. To the best of our knowledge, there is very scarce data on B vitamins in preterm human milk [56,57,58,59].

The spectral region δ 3.033–3.055, containing signals from creatine and creatinine, was found to be negatively correlated with the lactation stage. Visual inspection of NMR data allowed us to ascribe this result to a decrease in creatine content. This metabolite is essential for brain metabolism [60]. Similar to choline supply, infants receive their dietary creatine from the mother’s HM; however, due to low levels in HM, they must rely on endogenous synthesis [61]. This situation is particularly critical for preterm infants which become creatine-depleted in the early postnatal period [62].

To the best of our knowledge, no metabolomics studies thus far have examined the possible effect of GA length on the composition of the PT milk metabolome. Here, we observed a significant, although weak, impact of GA when comparing the two classes of samples taken from mothers with extremely and moderately preterm delivery. Our results showed that the levels of α1,3-linked fucosyl residues, 3′sialyllactoses, choline, myo-inositol, and glucosyl moieties in the metabolome of moderately preterm milk were higher than in the extremely preterm group. It is worth noting that these two groups significantly differ in both GA and in the type of delivery. Indeed, moderately premature infants were predominantly delivered by cesarean section, while the extremely premature ones were delivered mainly vaginally. Thus, our data interpretation could be influenced by this potential confounding factor. In the literature, there are limited data suggesting that mode of delivery may influence maternal HM composition, and not all of the results provided are in agreement. For instance, concerning HMO molecular components, several studies have not found any association between delivery mode and HMOs composition [63,64]. On the contrary, other investigations have pointed out significantly higher concentrations of lacto-*N*-tetraose and 6′SL (at day 30 post-partum) in women giving birth through C-section and significantly lower concentrations of some HMOs, including 2′FL, 3′SL, and LNFP III at day two compared to mothers giving birth by vaginal delivery [49]. Furthermore, a recent metabolomics investigation on term milk has pointed out significant differences by country in specific HM metabolites after vaginal or caesarean delivery, ascribed to possible differences in clinical procedures and antibiotic use [12]. It is worth noting that in our study the very premature infants were predominantly delivered by cesarean section, as were the moderately preterm ones. However, when the very preterm delivery group was compared to the extremely preterm delivery group, we did not observe significant differences due to prematurity (R^2^time = 0.72 (*p* = 0.01), Q^2^time = 0.37 (*p* = 0.01), R^2^prematurity = 0.64 (*p* = 0.61), Q^2^prematurity = 0.26 (*p* = 0.21)). Therefore, the delivery mode might be a confounding factor in this case; being significantly different for the two groups, we can affirm that the effect of delivery mode, if one exists, is not strong. Nevertheless, in light of the above-mentioned data in the literature, we cannot totally rule out a possible impact of the delivery mode on our findings; thus, in this context, our results are intended to be hypothesis generating. Although we are currently unable to explain the nature of these findings, defining the relation between the metabolic differences that we observed and the prematurity degree is essential for advancing the field of PT infant nutrition research.

We recognize several limitations to our study, first its small sample size and second its use of a single analytical platform. The exploitation of the combined strengths of ^1^H NMR spectroscopy and mass spectrometry can definitely improve the panels of detectable metabolites and thus provide a better interpretation of the biological function of preterm milk. The strength of this study is the presence of an almost complete set of samples collected a three time points and the diversification of specimens according to the degree of prematurity. We analyzed 97 preterm milk samples, provided by 36 women: 38 samples from 14 mothers delivering extremely preterm, 28 from 11 women delivering very preterm and 31 from 11 mothers delivering moderately preterm. To the best of our knowledge, this is the first metabolomics investigation on preterm HM to adopt this experimental design. Usually, in metabolomics-based pilot studies where multivariate data analysis approaches are applied, 10–15 observations for each group of interest are considered suitable to perform reliable data analysis. In our study, the number of observations at each time point for each group was in line with that used in other metabolomics pilot investigations [65,66,67]. Moreover, we applied a randomization test to check for overfitting and rejected the null hypothesis that metabolite concentration is independent of prematurity and time. As the *p*-values were less than 0.10, we can affirm that most likely both time and prematurity affect the metabolic profile of PT milk and thus we can reasonably affirm that our results cannot be due to random bias.

## 5. Conclusions

In this study, we have performed an analysis of the longitudinal composition of preterm HM metabolome taking into consideration the degree of prematurity as a discriminant factor. We observed that the more preponderant compositional variations were due to the mother’s phenotypes and the lactation stage. The major finding of our work is the evidence of significant, although weak, changes in the content of some metabolites in term of the GA length when comparing extremely and moderately preterm delivery groups. This observation raises questions about the biological significance of these differences. Due to the importance of nutrition in the early period of life of preterm infants, further studies on a new and larger cohort are warranted in order to validate our findings and to explore the implications of these differences on the health outcomes of preterm infants as well as on the optimal nutrition of these vulnerable patients.

## Figures and Tables

**Table 1:** Descriptive characteristics of mothers and infants, grouped according to the gestational maturity ^1^.

	Extremely Preterm (*n* = 14)	Very Preterm (*n* = 11)	Moderately Preterm (*n* = 11)
Mothers			
Maternal age, y (ANOVA, *p* = 0.48)	34.6 ± 5.1	34.6 ± 4.3	35.2 ± 3.4
Maternal BMI, kg/m^2^ (ANOVA, *p* = 0.41)	23.4 ± 3.9	22.9± 3.6	24.6 ± 6.9
Type of pregnancy (Singleton/Twins) (Chi-squared test, *p* = 0.69)	12/2	9/2	8/3
Mode of delivery (vaginal/casarean section) (Chi-squared test, *p* = 0.006)	10/4	2/9	2/9
**Infants**			
Gender (Male/Female) (Chi-squared test, *p* = 0.70)	6/10	6/7	5/9
Birth weight, g (ANOVA, *p* = 0.002)	977 ± 233	1382 ± 357	1369 ± 375
Gestational age, wk [min–max]	26 [23–28]	30 [29–31]	33 [32,33]
**Milk Samples**			
Colostrum (3−6 lactation days)	12	9	11
Transitional milk (7−15 lactation days)	14	11	11
Mature milk (16–26 lactation days)	12	8	9
**Lewis (Le) and Secretor (Se) phenotype of mothers**^2^ (Chi-square test, *p* = 0.33)			
Se^+^/Le^+^	11	9	6
Se^−^/Le^+^	1	1	4
Se^+^/Le^−^	2	1	1

^1^ Continuous normally distributed data are presented as means ± standard deviation, whereas categorical data are presented as the number of occurrences per level. ^2^ Secretor/Lewis blood group status was estimated according to the NMR fucosylated oligosaccharide profile of milk.

**Table 2:** LME modelling results for longitudinal data using the 44 features and all the three degrees of prematurity.

Integrated Region ^1^ (ppm)	Annotation ^2^	Coefficient ^3^	p[time] ^4^	BH ^5^	R^2^_total_^6^
4.051–4.077	myo-inositol	−2.5 × 10^−2^	8.5 × 10^−11^	1	6.6 × 10^−1^
4.632–4.650	glucosyl moiety	−1.3 × 10^−2^	9.2 × 10^−9^	1	9.7 × 10^−1^
4.515–4.548	galactose moiety in α1,2-linked Fuc	−4.3 × 10^−2^	1.5 × 10^−8^	1	8.1 × 10^−1^
4.203–4.274	α1,2-linked Fuc residues ^7^	−6.2 × 10^−2^	1.9 × 10^−8^	1	8.7 × 10^−1^
5.304–5.336	α1,2-linked Fuc residues ^8^	−5.2 × 10^−2^	2.6 × 10^−8^	1	8.9 × 10^−1^
4.278–4.322	α1,2-linked Fuc residues	−1.7 × 10^−2^	4.8 × 10^−8^	1	9.7 × 10^−1^
1.215–1.294	CH_3_ in Fuc(α1-2) ^9^	−2.6 × 10^−1^	1.2 × 10^−7^	1	8.9 × 10^−1^
5.220–5.254	lactose	5.1 × 10^−2^	2.1 × 10^−7^	1	8.6 × 10^−1^
3.274–3.322	lactose	8.3 × 10^−2^	3.8 × 10^−7^	1	8.2 × 10^−1^
3.190–3.198	choline	−6.6 × 10^−3^	4.4 × 10^−7^	1	7.1 × 10^−1^
3.033–3.055	creatine and creatinine	−2.7 × 10^−3^	8.6 × 10^−7^	1	2.9 × 10^−1^
5.181–5.210	glucosyl moieties	−1.6 × 10^−2^	1.8 × 10^−6^	1	8.8 × 10^−1^
1.467–1.498	alanine	6.2 × 10^−3^	3.1 × 10^−6^	1	5.1 × 10^−1^
0.926–0.941	pantothenate	−4.8 × 10^−3^	8.1 × 10^−6^	1	3.9 × 10^−1^
3.001–3.015	U	−8.9 × 10^−4^	2.0 × 10^−5^	1	6.1 × 10^−1^
3.455–3.522	U	−3.9 × 10^−2^	2.1 × 10^−5^	1	7.1 × 10^−1^
2.397–2.485	glutamine	5.7 × 10^−3^	2.1 × 10^−5^	1	5.4 × 10^−1^
2.648–2.703	citrate	−4.6 × 10^−2^	2.2 × 10^−5^	1	5.7 × 10^−1^
3.226–3.237	GPC	5.0 × 10^−2^	2.4 × 10^−5^	1	2.8 × 10^−1^
2.750–2.793	3′SL	−4.8 × 10^−3^	3.7 × 10^−5^	1	8.2 × 10^−1^
2.518–2.703 ^10^	citrate	−8.7 × 10^−2^	4.1 × 10^−5^	1	5.7 × 10^−1^
2.518–2.574	citrate	−4.1 × 10^−2^	8.5 × 10^−5^	1	5.8 × 10^−1^
2.331–2.385	glutamate	1.9 × 10^−2^	1.2 × 10^−4^	1	5.5 × 10^−1^
4.133–4.155	galactose moiety	−1.6 × 10^−2^	1.4 × 10^−4^	1	9.5 × 10^−1^
2.015–2.086	*N*-Acetylglucosammine	−1.4 × 10^−1^	1.9 × 10^−4^	1	8.4 × 10^−1^
1.691–1.781	3′SL, 6′SL	−1.1 × 10^−2^	2.8 × 10^−4^	1	9.0 × 10^−1^
3.124–3.177	U	2.4 × 10^−3^	3.7 × 10^−4^	1	7.9 × 10^−1^
8.368–8.453	U	−4.7 × 10^−3^	4.3 × 10^−4^	1	9.7 × 10^−1^
3.199–3.207	U	−6.4 × 10^−3^	5.5 × 10^−4^	1	7.5 × 10^−1^
0.890–0.941 ^11^	pantothenate	−1.1 × 10^−2^	6.4 × 10^−4^	1	3.9 × 10^−1^
0.945–0.979	leucine	−3.1 × 10^−3^	4.2 × 10^−3^	1	3.5 × 10^−1^
0.890–0.906	pantothenate	−5.8 × 10^−3^	9.6 × 10^−3^	1	3.9 × 10^−1^
4.156–4.173	galactose moieties	−1.2 × 10^−2^	1.9 × 10^−2^	1	8.9 × 10^−1^
5.277–5.296	α1,2-linked Fuc residues ^12^	−5.7 × 10^−3^	2.0 × 10^−2^	1	6.9 × 10^−1^
1.315–1.344	threonine	−1.5 × 10^−2^	7.8 × 10^−2^	0	4.1 × 10^−1^
3.215–3.225	phosphocholine	2.8 × 10^−2^	8.4 × 10^−2^	0	6.9 × 10^−1^
5.019–5.047	α1,4-linked Fuc residues	2.4 ×10^−3^	1.3 × 10^−1^	0	9.3 × 10^−1^
5.148–5.169	α1,2-linked Fuc residues ^13^	−1.4 × 10^−3^	1.9 × 10^−1^	0	9.6 × 10^−1^
0.980–1.002	valine	−4.3 × 10^−4^	3.0 × 10^−1^	0	2.6 × 10^−1^
1.032–1.057	valine	1.7 × 10^−4^	5.5 × 10^−1^	0	4.3 × 10^−1^
1.138–1.214	CH_3_ in α1,3-Fuc and α1,4-Fuc	−8.5 × 10^−3^	6.0 × 10^−1^	0	9.3 × 10^−1^
0.98–1.057 ^14^	valine	−2.7 × 10^−4^	7.0 × 10^−1^	0	3.3 × 10^−1^
5.371–5.415	α1,3-linked Fuc residues ^15^	−5.5 × 10^−4^	8.0 × 10^−1^	0	8.4 × 10^−1^
5.426–5.468	α1,3-linked Fuc residues ^15^	−5.2 × 10^−5^	9.8 × 10^−1^	0	8.4 × 10^−1^

^1^ Integration interval used to quantify the features. ^2^ Chemical meaning. ^3^ Coefficient of the fixed effect for time. ^4^ *p*-value. ^5^ 1 if the feature has been selected when controlling the false discovery rate using the Benjamini-Hochberg (BH) procedure at level δ = 0.05, and 0 if the feature has not been selected. ^6^ Explained total data variation. ^7^ 2′FL, LDFT. ^8^ 2′FL, LNFP I. ^9^ 2′FL, LNFP I, LNDFH I, LDFT. ^10^ Both doublets of citrate. ^11^ Both peaks of pantothenate. ^12^ LDFT; ^13^ LNDFH I; ^14^ Both doublets of valine; ^15^ 3FL, LNDFH II, LDFT. Abbreviations: 2′FL, 2′fucosyllactose; 3FL, 3fucosyllactose; GPC, glycero-3-phosphocholine; LDFT lactodifucotetraose; LNDFH I, lacto-*N*-difucohesaose I; LNDFH II, lacto-*N*-difucohesaose II; LNFP I, lacto-*N*-fucopentaose I; SL: sialyllactose; U, unknown.

## Data Availability

The data presented in this study are available on request to the corresponding author.

## Supplementary Materials

The following are available online at https://www.mdpi.com/article/10.3390/foods11030345/s1, Figure S1: Biplot of the PCA model built considering the binned spectra colored according to maternal HMOs; Figure S2: LME modelling of data from the milk samples of extremely and moderately preterm delivery groups, using the 44 features. The *p*-values of the coefficients of the fixed effects of prematurity and time are reported in the same plot as –log10 values. Dashed red lines are used to indicate the thresholds corresponding to *p* = 0.05. The meaning of the feature codes is reported in Table S2; Table S1: Descriptive characteristics of mothers delivering extremely and moderately preterm and those of their infants; Table S2: LME modelling results using the 44 features.
