# Supplementary material for: NMR Metabonomic Profile of Preterm Human Milk in the First Month of Lactation: From Extreme to Moderate Prematurity
Source: Foods. 2022 Jan 26;11(3):345. doi: 10.3390/foods11030345 (PMC8834565; doi:10.3390/foods11030345)
Supplement: Supplementary file 1 [file foods-11-00345-s001.zip › foods-1560605-supplementary.pdf]

SUPPLEMENTARY MATERIALS

NMR Metabonomic Profile of Preterm Human Milk in The First Month of Lactation: From Extremely To Moderate Prematurity

Chiara Peila[1], Stefano Sottemano [1], Flaminia Cesare Marincola [2*], Matteo Stocchero [3,4*], Nicoletta Grazia Pusceddu [1], Angelica Dessì [5], Eugenio Baraldi [3,4], Vassilios Fanos [5], Enrico Bertino [1]

[1] Neonatal Unit, University of Turin, City of Health and Science of Turin, 10126 Turin, Italy; enrico.bertino@unito.it (E.B.); chiara.peila@unito.it (C.P.); stefano.sottemano@unito.it (S.S.)
[2] Department of Chemical and Geological Sciences, Cittadella Universitaria di Monserrato, University of Cagliari, 09042 Monserrato, Cagliari, Italy; flaminia@unica.it (F.C.M.); nicoletta.labor@gmail.com (N.G.P.)
[3] Department of Women's and Children's Health, University of Padova, 35128 Padova, Italy; eugenio.baraldi@unipd.it (E.B.); matteo.stocchero@unipd.it (M.S.).
[4] Institute of Pediatric Research (IRP), Fondazione Città della Speranza, 35128 Padova, Italy
[5] Neonatal Intensive Care Unit, Neonatal Pathology and Neonatal Section, Azienda University Polyclinic, University of Cagliari, 09042 Cagliari, Italy; angelicadessi@unica.it (A.D.); vafanos@tin.it (V.F.)

*Correspondence: flaminia@unica.it; matteo.stocchero@unipd.it

Combining PLS2 and LME modelling to investigate longitudinal data from a multivariate point of view

Given the matrix of the measured variables $Y = [y_1 ... y_P]$ and the design matrix of the fixed effects X_{fixed}, the objective is to decompose Y as

$$Y = Y_{fixed} + Y_{random} + F \tag{1}$$

where the data variation in Y_{fixed} is explained by X_{fixed}, the data variation in Y_{random} is associated to the random effects described by a suitable design matrix X_{random} and the residual matrix F is the part of Y that is not related to the fixed factors or to the random effects. The following two-step procedure is used to solve the problem:

1- LME modelling is applied to model each single measured variable, i.e. each column of Y, by

$$y_p = X_{fixed}\beta_p + X_{random}u_p + \sigma_p \quad \text{with} \quad p = 1, ..., P \tag{2}$$

where $u_p \sim N(\underline{0}, G)$ is a random vector specifying the coefficients of the random effects (it is assumed to be multi-normally distributed with mean $\underline{0}$ and covariance matrix G) and $\sigma \sim N(\underline{0}, \sigma^2 I)$ is the error term. The random parts $X_{random}u_p$ calculated for the different variables are juxtaposed to obtain the matrix

$$Y_{random} = [X_{random}u_1 ... X_{random}u_P] = X_{random}U_{random} \tag{3}$$

being $U_{random} = [u_1 ... u_P]$.

2- The matrix $Y - Y_{random}$ is decomposed using PLS2 [1]. Specifically, the regression model

$$X_{fixed} = (Y - Y_{random})B_A + E_A \tag{4}$$

is considered. The matrix of the regression coefficients is B_A and the residual matrix is E_A. Stopping the PLS2 algorithm after A iterations and post-transforming the model, one obtains

$$Y - Y_{random} = TP^t + T_o P_o^t + F_A \tag{5}$$

where TP^t is the scores by loadings block that explains the matrix of the fixed effects, $T_o P_o^t$ is the scores by loadings block that includes the data variation of $Y - Y_{random}$ captured by the PLS2 model orthogonal to X_{fixed} and F_A is the part of $Y - Y_{random}$ that is not used in the regression model. As a result, the following matrix decomposition is obtained

$$Y = TP^t + Y_{random} + T_o P_o^t + F_A. \tag{6}$$

The problem is solved once the following two matrices are introduced

$$Y_{fixed} = TP^t \tag{7}$$

$$F = T_o P_o^t + F_A. \tag{8}$$

The columns of the matrix T are the scores generated by the model. They are considered the basis to build the latent factors that may be explained in terms of the fixed factors. The possibility to obtain latent factors that can be fully explained in terms of specific experimental factors depends on the properties of the design matrix $\mathsf{X}_{\text{fixed}}$ and on the goodness-of-fit of the PLS2 model. Indeed, the scores explain all the experimental factors considered in $\mathsf{X}_{\text{fixed}}$ at the same time and, in general, each factor is not associated to a single score but to a combination of scores. To obtain latent factors that can be easily interpreted in terms of fixed factors, we suggest to apply procrustes analysis to rotate the score space.

Cross-validation and permutation testing are applied to assess the relevance of the fixed factors and stability selection [2] used to identified the subset of measured variables that is mainly associated with the fixed factors.

References

[1] Stocchero, M. Iterative deflation algorithm, eigenvalue equations, and PLS2. *J. Chemometr.* **2019**, *33*, e3144.

[2] Stocchero, M. Relevant and irrelevant predictors in PLS2. *J. Chemometr.* **2020**, *34*, e3237.

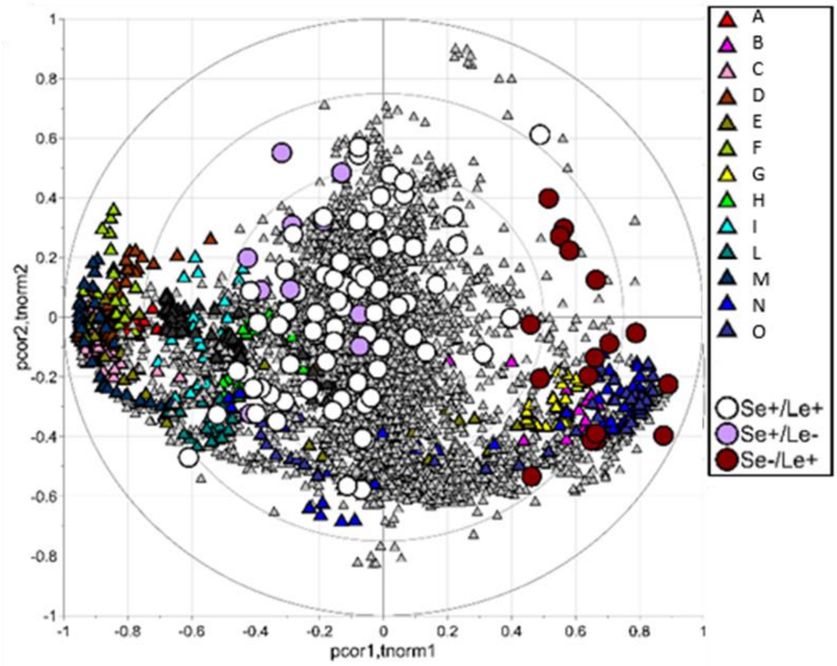

Figure S1. Biplot of the PCA model built considering the binned spectra, colored according to maternal HMOs phenotype (Se^+/Le^+ in white; Se^+/Le^- in violet; Se^-/Le^+ in red); measured features (bins) are reported as gray triangles or colored triangles in the case of HMOs signals. Abbreviations: A,B,E: galactose moieties; C, D, M: α1,2-linked Fuc residues; F, I: glucosyl moiety; G: α1,4-linked Fuc residues; H, L, N, O: α1,3-linked Fuc residues.

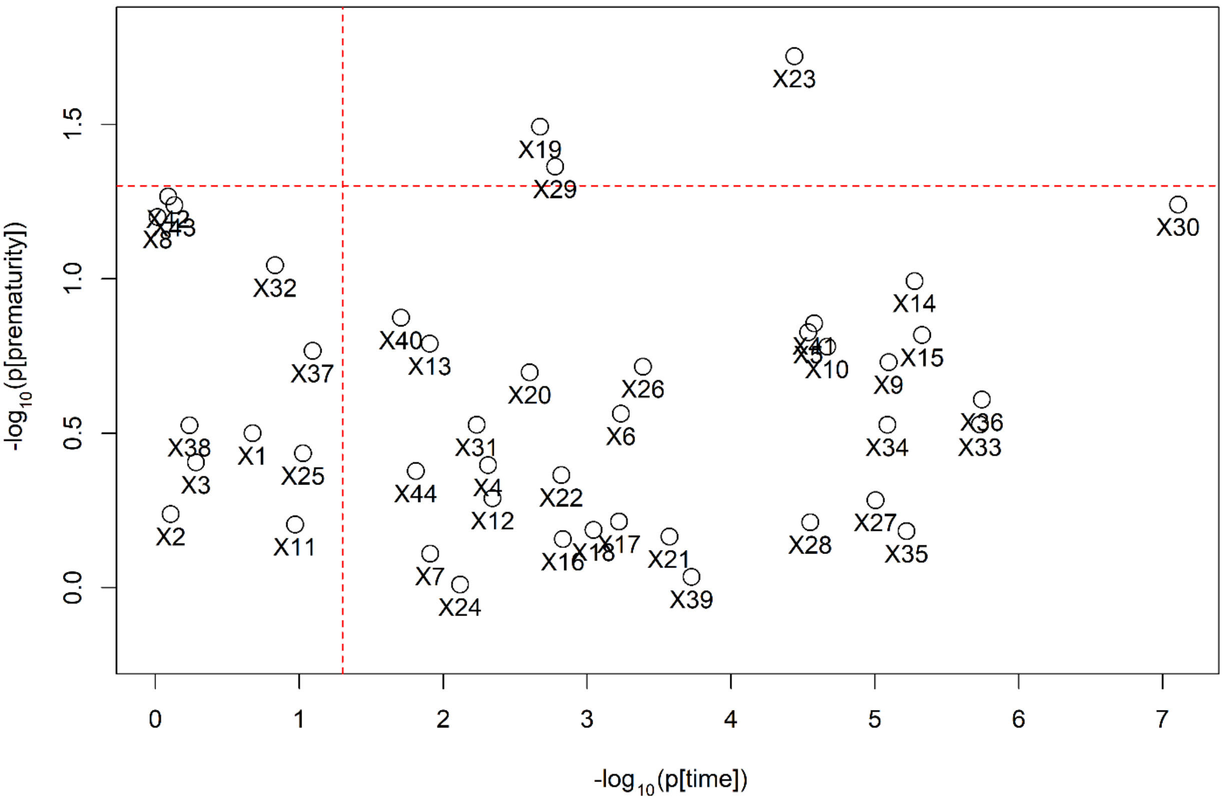

Figure S2. LME modelling of data from the milk samples of extremely and moderately preterm delivery groups, using the 44 features. The p values of the coefficients of the fixed effects of prematurity and time are reported in the same plot as $-\log_{10}$ values. Dashed red lines are used to indicate the thresholds corresponding to $p = 0.05$. The meaning of the feature codes is reported in Table S2.

Table S1. Descriptive characteristics of mothers delivering extremely and moderately preterm and those of their infants [1].

	**Extremely preterm** (n = 14)	**Moderately preterm** (n = 11)
**Mothers**		
Maternal age, y (ANOVA, $p = 0.74$)	34.6 ± 5.1	35.2 ± 3.4
Maternal BMI, kg/m^2 (ANOVA, $p = 0.59$)	23.4 ± 3.9	24.6 ± 6.9
Type of pregnancy (Singleton/Twins) (Fisher's exact $p = 0.62$)	12/2	8/3
Mode of delivery (vaginal/casarean section) (Fisher's exact $p = 0.015$)	10/4	2/9
**Infants**		
Gender (Male/Female) (Fisher's exact $p = 1.00$)	6/10	5/9
Birth weight, g (ANOVA $p = 0.002$)	977 ± 233	1369 ± 375
Gestational age, wk [min-max]	26 [23-28]	33 [32-33]
**Milk samples**		
Colostrum	12	11
Transitional milk	14	11
Mature milk	12	9
**Lewis (Le) and Secretor (Se) phenotype of mothers** [2] (Chi-squared test, $p = 0.19$)		
Se^+/Le^+	11	6
Se^-/Le^+	1	4
Se^+/Le^-	2	1

[1] Continuous normally distributed data are presented as means ± standard deviation whereas categorical data as number of occurrences per level. [2] Secretor/Lewis blood group status was estimated according to NMR fucosylated oligosaccharides profile of milk.

Table S2. LME modelling results using the 44 features

Integrated region [1] (ppm)	Annotation [2]	code	Coeff. time [3]	Coeff. prematurity [4]	p[time] [5]	p[prematurity] [5]	R^2total [6]
3.190-3.198	choline	X23	-5.5×10^{-3}	3.7×10^{-3}	3.6×10^{-5}	1.9×10^{-2}	7.2×10^{-1}
2.750-2.793	3′SL	X19	-4.1×10^{-3}	6.6×10^{-3}	2.1×10^{-3}	3.2×10^{-2}	7.7×10^{-1}
3.455-3.522	U	X29	-3.5×10^{-2}	4.8×10^{-2}	1.7×10^{-3}	4.3×10^{-2}	7.2×10^{-1}
5.371-5.415	α1,3-linked Fuc residues	X42	5.4×10^{-4}	1.3×10^{-2}	8.2×10^{-1}	5.4×10^{-2}	9.1×10^{-1}
4.051-4.077	myo-insositol	X30	-2.4×10^{-2}	1.4×10^{-2}	7.8×10^{-8}	5.7×10^{-2}	7.1×10^{-1}
5.426-5.468	α1,3-linked Fuc residues	X43	9.8×10^{-4}	1.8×10^{-2}	7.4×10^{-1}	5.8×10^{-2}	9.2×10^{-1}
1.138-1.214	CH_3 in α1,3-Fuc and α1,4-Fuc	X8	7.8×10^{-4}	2.0×10^{-1}	9.7×10^{-1}	6.3×10^{-2}	9.3×10^{-1}
4.156-4.173	galactose moietiesies	X32	-9.7×10^{-3}	3.1×10^{-2}	1.5×10^{-1}	9.0×10^{-2}	9.4×10^{-1}
2.331-2.385	glutamate	X14	2.5×10^{-2}	-1.3×10^{-2}	5.3×10^{-6}	1.0×10^{-1}	6.0×10^{-1}
5.277-5.296	α1,2-linked Fuc residues	X40	-5.1×10^{-3}	-6.6×10^{-3}	2.0×10^{-2}	1.3×10^{-1}	6.7×10^{-1}
5.304-5.336	α1,2-linked Fuc residues	X41	-5.3×10^{-2}	-5.2×10^{-2}	2.6×10^{-5}	1.4×10^{-1}	9.2×10^{-1}
0.926-0.941	pantothenate	X5	-4.3×10^{-3}	1.8×10^{-3}	2.9×10^{-5}	1.5×10^{-1}	4.3×10^{-1}
2.397-2.485	glutamine	X15	6.9×10^{-3}	3.2×10^{-3}	4.7×10^{-6}	1.5×10^{-1}	6.1×10^{-1}
2.015-2.086	*N*-Acetylglucosammine	X13	-1.1×10^{-1}	1.2×10^{-1}	1.2×10^{-2}	1.6×10^{-1}	8.3×10^{-1}
1.467-1.498	alanine	X10	7.1×10^{-3}	-2.9×10^{-3}	2.1×10^{-5}	1.7×10^{-1}	8.7×10^{-1}
5.019-5.047	α1,4-linked Fuc residues	X37	3.5×10^{-3}	1.4×10^{-2}	8.1×10^{-2}	1.7×10^{-1}	9.3×10^{-1}
1.235-1.294	α1,2-linked Fuc residues	X9	-2.7×10^{-1}	-2.8×10^{-1}	8.0×10^{-6}	1.9×10^{-1}	8.9×10^{-1}
3.226-3.237	glycero-3-phosphocholine	X26	5.1×10^{-2}	-2.3×10^{-2}	4.1×10^{-4}	1.9×10^{-1}	3.7×10^{-1}
3.001-3.015	U	X20	-7.3×10^{-4}	-4.7×10^{-4}	2.5×10^{-3}	2.0×10^{-1}	5.1×10^{-1}

4.632-4.650	glucosyl moiety	X36	-1.4×10^{-2}	-1.2×10^{-2}	1.8×10^{-6}	2.5×10^{-1}	9.7×10^{-1}
0.890-0.941	pantothenate	X6	-9.1×10^{-3}	3.5×10^{-3}	5.8×10^{-4}	2.7×10^{-1}	3.4×10^{-1}
4.203-4.274	α1,2-linked Fuc residues	X33	-6.5×10^{-2}	-4.3×10^{-2}	1.9×10^{-6}	3.0×10^{-1}	8.6×10^{-1}
4.133-4.155	galactose moiety	X31	-1.5×10^{-2}	-1.6×10^{-2}	5.8×10^{-3}	3.0×10^{-1}	9.4×10^{-1}
4.278-4.322	α1,2-linked Fuc residues	X34	-1.7×10^{-2}	-1.3×10^{-2}	8.2×10^{-6}	3.0×10^{-1}	9.7×10^{-1}
5.148-5.169	α1,3-linked Fuc residues	X38	-6.6×10^{-4}	-7.8×10^{-3}	5.8×10^{-1}	3.0×10^{-1}	9.5×10^{-1}
0.980-1.002	valine	X1	-6.0×10^{-4}	-6.3×10^{-4}	2.1×10^{-1}	3.2×10^{-1}	3.0×10^{-1}
3.215-3.225	phosphocholine	X25	3.4×10^{-2}	-2.5×10^{-2}	9.4×10^{-2}	3.7×10^{-1}	8.1×10^{-1}
0.980-1.057	valine	X3	-5.0×10^{-4}	-8.9×10^{-4}	5.2×10^{-1}	3.9×10^{-1}	3.3×10^{-1}
0.890-0.906	pantothenate	X4	-4.8×10^{-3}	1.7×10^{-3}	4.9×10^{-3}	4.0×10^{-1}	2.5×10^{-1}
8.368-8.453	U	X44	-3.9×10^{-3}	-4.5×10^{-3}	1.6×10^{-2}	4.2×10^{-1}	9.6×10^{-1}
3.124-3.177	U	X22	3.0×10^{-3}	-7.4×10^{-4}	1.5×10^{-3}	4.3×10^{-1}	7.8×10^{-1}
1.691-1.781	3′SL, 6′SL	X12	-1.1×10^{-2}	4.1×10^{-3}	4.5×10^{-3}	5.1×10^{-1}	8.6×10^{-1}
3.274-3.322	lactose	X27	8.0×10^{-2}	-2.1×10^{-2}	9.9×10^{-6}	5.2×10^{-1}	7.0×10^{-1}
1.032-1.057	valine	X2	9.0×10^{-5}	-2.5×10^{-4}	7.8×10^{-1}	5.8×10^{-1}	4.2×10^{-1}
2.648-2.703	citrate	X17	-4.4×10^{-2}	-1.1×10^{-2}	6.0×10^{-4}	6.1×10^{-1}	7.0×10^{-1}
5.220-5.254	lactose	X28	4.7×10^{-2}	-8.7×10^{-3}	2.8×10^{-5}	6.1×10^{-1}	7.8×10^{-1}
1.315-1.344	threonine	X11	-1.2×10^{-2}	4.2×10^{-3}	1.1×10^{-1}	6.2×10^{-1}	4.3×10^{-2}
2.518 -2.706	citrate	X18	-8.3×10^{-2}	-1.9×10^{-2}	9.0×10^{-4}	6.5×10^{-1}	7.0×10^{-1}
4.515-4.548	galactose moiety in α1,2-linked Fuc	X35	-4.4×10^{-2}	-9.0×10^{-3}	6.0×10^{-6}	6.6×10^{-1}	8.1×10^{-1}
3.033-3.055	creatine and creatinine	X21	-2.4×10^{-3}	-2.8×10^{-4}	2.7×10^{-4}	6.8×10^{-1}	1.9×10^{-1}
2.518-2.574	citrate	X16	-3.9×10^{-2}	-8.1×10^{-3}	1.5×10^{-3}	7.0×10^{-1}	7.0×10^{-1}

0.945-0.979	leucine	X7	-3.2×10^{-3}	4.6×10^{-4}	1.2×10^{-2}	7.8×10^{-1}	2.9×10^{-1}
5.181-5.210	glucosyl moieties	X39	-1.5×10^{-2}	9.2×10^{-4}	1.9×10^{-4}	9.2×10^{-1}	8.8×10^{-1}
3.199-3.207	U	X24	-5.7×10^{-3}	1.2×10^{-4}	7.6×10^{-3}	9.8×10^{-1}	6.7×10^{-1}

[1] Integration interval used to quantify the features. [2] Chemical meaning. [3] Coefficient of the fixed effect for time. [4] Coefficient of the fixed effect for preamaturity. [5] p-value. [6] Explained total data variation.

U, unknown.
